# A spatial interaction incorporated betweenness centrality measure

**DOI:** 10.1371/journal.pone.0268203

**Published:** 2022-05-20

**Authors:** Xiaohuan Wu, Wenpu Cao, Jianying Wang, Yi Zhang, Weijun Yang, Yu Liu

**Affiliations:** 1 Institute of Remote Sensing and Geographical Information Systems, School of Earth and Space Sciences, Peking University, Beijing, China; 2 Guangzhou Urban Planning & Design Survey Research Institute, Guangzhou, China; 3 State Key Laboratory of Media Convergence Production Technology and Systems, Beijing, China; China University of Geosciences, CHINA

## Abstract

Betweenness centrality (BC) is widely used to identify critical nodes in a network by exploring the ability of all nodes to act as intermediaries for information exchange. However, one of its assumptions, i.e., the contributions of all shortest paths are equal, is inconsistent with variations in spatial interactions along these paths and has been questioned when applied to spatial networks. Hence, this paper proposes a spatial interaction incorporated betweenness centrality (SIBC) for spatial networks. SIBC weights the shortest path between each node pair according to the intensity of spatial interaction between them, emphasizing the combination of a network structure and spatial interactions. To test the rationality and validity of SIBC in identifying critical nodes and edges, two specific forms of SIBC are applied to the Shenzhen street network and China’s intercity network. The results demonstrate that SIBC is more significant than BC when we also focus on the network functionality rather than only on the network structure. Moreover, the good performance of SIBC in robustness analysis illustrates its application value in improving network efficiency. This study highlights the meaning of introducing spatial configuration into empirical models of complex networks.

## Introduction

A spatial network is a graph in which nodes and edges are embedded in a geometric space equipped with a certain metric [1]. Understanding the properties of spatial networks is essential for characterizing the structure and functionality of many complex systems, such as biological and transportation systems [2, 3]. Because of their capability to interpret the static features and simulate the dynamic mechanisms of complex systems, various network analysis methods have been applied to spatial networks [4, 5].

Among numerous network analysis measures, betweenness centrality (BC) plays a vital role in identifying central nodes or edges and analyzing the global structure of a network [6]. Derived from the social science, BC measures the extent to which a node or an edge works as an intermediary in global information exchanges across a social network. In other words, it can indicate which nodes or edges information is more likely to pass through. BC has been extended to analyze the spatial network structure and functionality in many studies; for example, the evolution of street networks [3, 7], the structural invariants in street networks [8], the prediction of urban traffic flows [9, 10], the prediction and predictability of global epidemics [2], and the identification of critical locations in spatial networks [11, 12].

However, conventional BC, designed for social networks, only considers the network topology and does not involve spatial elements. Regarding spatial networks, the topology alone can only provide partial information, and the network structure largely depends on the geometric space (i.e., node locations and edge lengths) [1]. Moreover, spatial interactions between all node pairs are critical for understanding the functionality of spatial networks. When conventional BC is applied to explain the structure and functionality of spatial networks, the results are often unsatisfactory because the edge lengths and the spatially variant interaction demands are ignored [13, 14].

Specifically, conventional BC makes two assumptions about information exchanges in social networks: (1) information is transmitted via the shortest paths identified from the network topology, and (2) all node pairs exchange the same amount of information [8]. As conventional BC is applied to various types of networks, these assumptions have been questioned and refined [13, 15]. The most common modification has been to calculate the edge-weighted shortest path in an edge-weighted network. The edge weight (called the edge cost in this paper to distinguish it from the weight of the shortest path) denotes the cost of passing along an edge, which is often represented by the edge length [16, 17]. BC can therefore not only be used for unweighted networks, such as social networks, but also for edge-weighted networks, such as spatial networks. For spatial network analyses, considering edge-weighted shortest paths can make BC embody the influence of the edge cost on the path choice so that the centrality of nodes and edges can be validly measured when we only concern the network structure.

Nevertheless, the above-mentioned modification still retains the second assumption. The varying interaction demands are still overlooked, leading to the importance of nodes or edges carrying large traffic volumes but passing through by a few shortest paths can be underestimated in a spatial network. A few studies have been published on modifying the second assumption. Borgatti and Everett [18] proposed a BC variant that weights all shortest paths inversely in proportion to their lengths. Hanzelka et al. [19] and Giustolisi et al. [20] verified the efficacy of weighting the shortest paths with the attributes of the end nodes. However, the weights of shortest paths in these studies still can not fully reflect the various interaction intensities. The former introduces the distance decay effect, while the latter considers the relationship between end nodes. These are insufficient when the research focuses on the effects of nodes and edges on the functionality of spatial networks. Therefore, it is meaningful to give a clear explanation of the variation in spatial interactions in BC when applying it to spatial problems [21].

As the application of BC in traffic flow estimation attracts much attention, some studies have recognized the important role of the spatial interaction in improving the ability of BC to estimate traffic flow [22–24]. In [22, 23], the authors verified that considering the actual measured origin-destination (OD) matrix in the conventional BC can get a better correlation between the BC and the traffic flow. In addition, the simulated spatial interaction was introduced into BC to estimate pedestrian flow on sidewalks [24]. All of these studies support the idea of this paper to introduce the spatial interaction into BC. However, traffic flow estimation is only one of the applications of BC. A comprehensive understanding of the impact of spatial interactions on the fundamental function of BC (i.e., measuring the centrality of nodes and edges) is still insufficient. Moreover, considering the importance of introducing the spatial interaction into BC, the application effects of the extended BC to more scenarios besides traffic flow estimation, such as hub node identification and robustness analysis, should also be worth analyzing.

Therefore, this paper proposes a general definition of spatial interaction incorporated betweenness centrality (SIBC), which is a combination of topologies, edge costs, and the intensity of spatial interactions. The topology and the edge cost determine the shortest paths in a network. The intensity of spatial interactions determines the contribution of each path to the centrality of the nodes or the edges it passes through or along. Depending on the methods of measuring spatial interactions, SIBC can be expressed in different forms. In this paper, we develop two specific forms to explore the impact of the spatial interaction on identifying critical nodes and edges in spatial networks: the observed OD flow incorporated betweenness centrality (SIBC_o_) and the gravity model incorporated betweenness centrality (SIBC_g_). The Shenzhen street network and China’s intercity network were used to verify the effectiveness of these measures. The spatial distributions of BC, SIBC_o_, and SIBC_g_ were compared to explore the different performances of these measures. Correlations between the traffic volume on streets and the three measures were analyzed to quantify the extent to which the SIBC is more suitable to evaluate the centrality of edges than BC in spatial networks. A robustness experiment investigated the impact of removing nodes or edges with high BC or high SIBC on a network, exploring the significance of SIBC in robustness analysis. Moreover, it is worth noting that the philosophy behind SIBC, that is, considering the spatial configuration of a network, also applies to common social networks. Therefore, we extended this idea to social networks and applied SIBC_g_ to the Florence families network to verify the validity of the extension.

## Methods

### Betweenness centrality

BC was designed to evaluate the ability of a node or an edge to control information exchanges or material flows in networks [6, 16]. To extend it to various applications, many BC variants have been proposed, such as random-walk betweenness centrality [25], communication betweenness centrality [26], and randomized shortest paths betweenness centrality [15]. BC is defined as the probability of a node or an edge acting as an intermediary between all the shortest paths in a network. Given a graph *G*(*V*, *E*), *V* is the set of nodes, and *E* is the set of edges, where |*V*| = *n*, |*E*| = *m*. The calculation for the BC of node *v* is expressed as follows:

$$\begin{array}{c} BC\left(v\right)=\sum_{s,t\in V;s\neq t\neq v}\frac{\sigma_{s,t}\left(v\right)}{\sigma_{s,t}} \end{array}$$ (1)

where *BC*(*v*) is the BC of node *v*, while *σ*_*s*,*t*_ and *σ*_*s*,*t*_(*v*) denote the total number of shortest paths and the number of shortest paths passing through node *v*, respectively, for the couple of nodes *s* and *t*.

Similarly, the BC of an edge *e* is the probability for all shortest paths in the network traversing it. The formulation is:

$$\begin{array}{c} BC\left(e\right)=\sum_{s,t\in V;s\neq t}\frac{\sigma_{s,t}\left(e\right)}{\sigma_{s,t}} \end{array}$$ (2)

where *σ*_*s*,*t*_(*e*) denotes the total number of shortest paths passing through edge *e* for the couple of nodes *s* and *t*.

Note that, BC can apply to both nodes and edges. The BC for nodes can be used to identify the hubs in a network, while the BC for edges can be used to find the ‘bridges’ in a network. Additionally, the above definitions can apply to both unweighted and edge-weighted networks. In unweighted networks, more than one shortest path might exist between each couple *s* and *t*. In edge-weighted networks, a single shortest path generally exists for a pair of nodes *s* and *t* and the fraction in Eqs 1 and 2 vanishes. Besides, if all nodes are reachable in a network, there are (*n* − 1)(*n* − 2) paths that might pass through node *v* when *v* is not the beginning or end of a path. We normalize the BC values of nodes by 2/((*n* − 1)(*n* − 2)) for undirected networks, and 1/((*n* − 1)(*n* − 2)) for directed networks. Similarly, the BC values of edges are normalized by 2/((*n* − 1)*n*) for undirected networks, and 1/((*n* − 1)*n*) for directed networks.

### Spatial interaction incorporated betweenness centrality

Here, we introduce spatial interactions into BC. When computing BC, each shortest path between two generic nodes *s* and *t* contributes a unitary increment to the BC of the traversed nodes or edges. After identifying the shortest paths between all pairs of nodes *s* and *t*, the BC of a node or an edge depends on the number of paths it is passed through or along. Hence, the BC values only depend on the network topology and the edge cost (for edged-weighted networks), which are used to identify all the shortest paths in a network.

However, topology and the edge cost only represent the structural characteristics of networks. In spatial networks, the spatial interaction describes the flow between each node pair. The distribution of spatial interactions in the network implies the transportation function of the nodes and edges in a network. If the calculation of BC does not consider spatially various interaction demands, it can only identify the central nodes or edges in terms of network structure, but cannot reflect the importance of nodes and edges in the network functionality. Therefore, it is meaningful to weight the shortest path between *s* and *t* with *f*(*s*, *t*) which denotes the intensity of spatial interactions between *s* and *t*. In this way, the centrality of nodes or edges traversed by the shortest path between *s* and *t* increases by a quantity *f*(*s*, *t*). Consequently, the formulations of the spatial interaction incorporated betweenness centrality (SIBC) are

$$\begin{array}{c} SIBC\left(v\right)=\sum_{s,t\in V;s\neq t\neq v}\frac{\sigma_{s,t}\left(v\right)}{\sigma_{s,t}}f\left(s,t\right) \end{array}$$ (3)

$$\begin{array}{c} SIBC\left(e\right)=\sum_{s,t\in V;s\neq t}\frac{\sigma_{s,t}\left(e\right)}{\sigma_{s,t}}f\left(s,t\right) \end{array}$$ (4)

**Fig 1:**
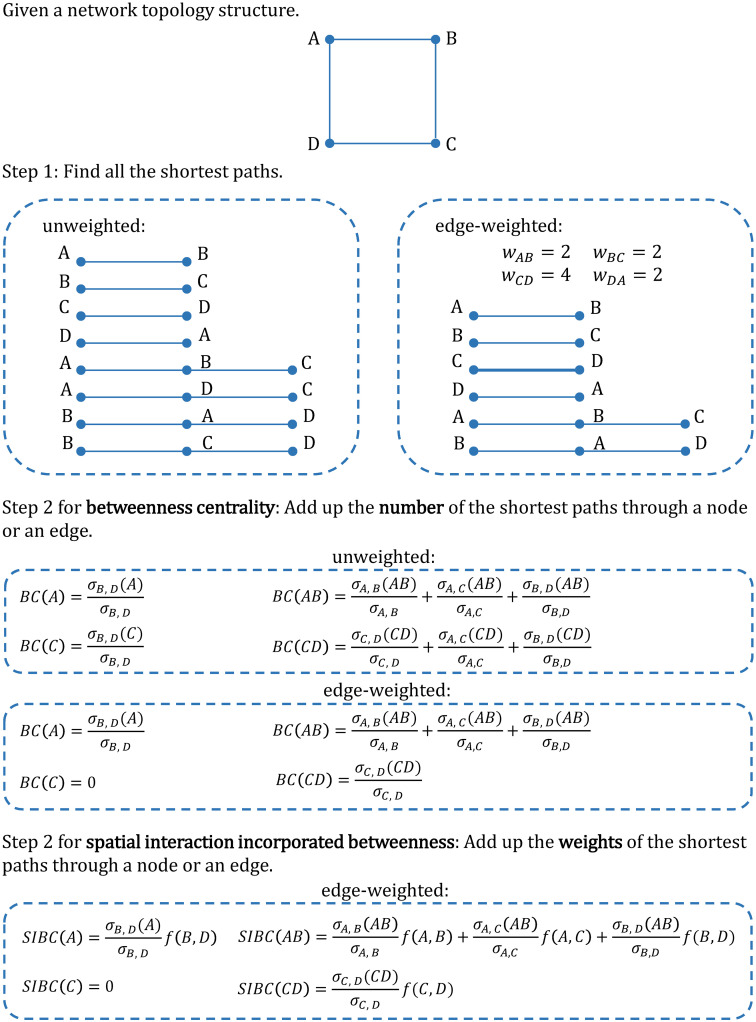
An example network. Comparison of the BC and SIBC calculations in the example network, taking nodes *A* and *C* and edges *AB* and *CD* as illustrative examples.

The calculation of SIBC involves two steps:

1. Find all the shortest paths according to the network topology and the edge cost, which is shown on the right of Step #1 in Fig 1.
2. Sum the number of spatial interactions carried by all the shortest paths through node *v* or along edge *e* according to Eqs 3 or 4, which is shown in Step #2 for SIBC in Fig 1.

We use the simplified network in Fig 1 to illustrate the difference between BC and SIBC and compare the different roles of the edge cost and the intensity of spatial interactions in the SIBC calculation. Here, the calculation of BC in both an unweighted and an edge-weighted network is considered. The SIBC only considers the edge-weighted case because the spatial network is necessarily an edge-weighted network. Step #1 for calculating the BC in an unweighted network is to find the path with the fewest edges for each node pair. Step #1 for calculating the BC in an edge-weighted network is the same as that for calculating the SIBC. In this step, the edge length representing the edge cost affects the identification of the shortest paths. When identifying the shortest paths in edge-weighted networks, the edge with the lowest cost will be selected first. As shown in Fig 1, the shortest paths “*A*–*D*–*C*” and “*B*–*C*–*D*” in the unweighted case are excluded after considering the edge cost. In Step #2, BC simply sums the number of shortest paths through a node or along an edge. SIBC sums the number of spatial interactions carried by the shortest paths through a node or along an edge. If the number of spatial interactions between all node pairs is 1, then the SIBC and BC are equivalent.

The difference between SIBC and BC is mainly reflected in Step #2. In this step, considering the number of spatial interactions causes different shortest paths to contribute differently to the centrality of nodes or edges. The SIBC values are determined by combining topologies, edge lengths, and the intensity of spatial interactions. For example, in the edge-weighted case in Fig 1, only one shortest path passes along edge *CD*, while three shortest paths pass along edge *AB*. When only considering the network structure, the BC of edge *AB* is higher than that of edge *CD*; however, in the SIBC calculation, if *f*(*C*, *D*) = 3, *f*(*A*, *B*) = *f*(*A*, *C*) = *f*(*B*, *D*) = 1, then *SIBC*(*CD*) = *SIBC*(*AB*). The results for SIBC imply that the traffic volume along edge *CD* and edge *AB* is the same after combining network structure and the intensity of spatial interactions.

For algorithm implementation, the shortest paths are obtained by using the standard Dijkstra algorithm [27], and the computation of SIBC uses an adapted Brandes algorithm [28]. Similar to the BC, if all nodes are reachable in a network, $\sum_{s,t\in V;s\neq t\neq v}f\left(s,t\right)$ or $\sum_{s,t\in V;s\neq t}f\left(s,t\right)$ traffic might transmit node *v* or edge *e*, respectively. Hence, the SIBC values of nodes are normalized by $2/\sum_{s,t\in V;s\neq t\neq v}f\left(s,t\right)$ for undirected networks, and $1/\sum_{s,t\in V;s\neq t\neq v}f\left(s,t\right)$ for directed networks. The SIBC values of edges are normalized by $2/\sum_{s,t\in V;s\neq t}f\left(s,t\right)$ for undirected networks, and $1/\sum_{s,t\in V;s\neq t}f\left(s,t\right)$ for directed networks.

### Two specific forms of spatial interaction incorporated betweenness centrality

When calculating SIBC, the intensity of spatial interactions between all node pairs is required. In general, the intensity of spatial interactions can be represented by the observed OD flow, that is, *f*(*s*, *t*) = *OD*_*s*,*t*_, like the form in [22, 23]. In this case, the formulations of SIBC can be expressed as:

$$\begin{array}{c} SIBC_{o}\left(v\right)=\sum_{s,t\in V;s\neq t\neq v}\frac{\sigma_{s,t}\left(v\right)}{\sigma_{s,t}}OD_{s,t} \end{array}$$ (5)

$$\begin{array}{c} SIBC_{o}\left(e\right)=\sum_{s,t\in V;s\neq t}\frac{\sigma_{s,t}\left(e\right)}{\sigma_{s,t}}OD_{s,t} \end{array}$$ (6)

where the *OD*_*s*,*t*_ denotes the observed OD flow between *s* and *t*.

However, in some application scenarios, the spatial interaction data may be incomplete or the cost of data acquisition too high. To avoid an unavailable SIBC because of insufficient data, we provide a gravity model incorporated betweenness centrality as another representation of SIBC. The gravity model can estimate the spatial interaction between two distinct locations according to their local attributes and the distance decay effect [29]. This model is widely used in human migration [30, 31], transport planning [32], disease spread [33], and spatial economics [34]. The formulations of the gravity model incorporated betweenness centrality are:

$$\begin{array}{c} SIBC_{g}\left(v\right)=\sum_{s,t\in V;s\neq t\neq v}\frac{\sigma_{s,t}\left(v\right)}{\sigma_{s,t}}\frac{m_{s}m_{t}}{d_{st}^{\beta }} \end{array}$$ (7)

$$\begin{array}{c} SIBC_{g}\left(e\right)=\sum_{s,t\in V;s\neq t}\frac{\sigma_{s,t}\left(e\right)}{\sigma_{s,t}}\frac{m_{s}m_{t}}{d_{st}^{\beta }} \end{array}$$ (8)

where *m*_*s*_ and *m*_*t*_ denote the number of trips leaving *s* and the number of trips arriving at *t*. The constant in the gravity model is ignored in Eqs 7 and 8 because it does not substantially affect the calculation results of the SIBC_g_. *d*_*st*_ is the shortest path length between *s* and *t*, and *β* denotes the distance friction coefficient used to quantify the influence of distance on spatial interactions. For different spatial conditions, the extent of the distance decay effect varies. Different fitting methods for the *β* have been given in many studies [35–37].

Note that, accompanied by estimating the intensity of spatial interactions, the gravity model introduces node attributes and the distance decay effect into BC. Node attributes can not only describe the features of nodes but also reflect the relationship between nodes. The distance between two nodes is inversely proportional to the attraction between two nodes and the probability of choosing the shortest path. It is easy to understand that the centrality of a location that connects two crucial nearby places should be more significant than that of a location that connects two negligible and distant places. Both of them can influence the centrality of nodes or edges independently regardless of interaction intensities [19, 20]. Therefore, the gravity model incorporated betweenness centrality can make the concept of SIBC more fundamental.

## Case studies

To verify the effectiveness and applicability of SIBC spatial networks, we applied its two forms to the Shenzhen street network and China’s intercity network based on the railway. The Shenzhen street network plays an essential role in urban planning and human activities [38]. China’s intercity network based on the railway is frequently used to explore the impact of rail lines on the centrality of cities [39, 40]. We analyzed the correlation between the traffic volume on each street and the three betweenness measures to quantitatively compare BC, SIBC_o_, and SIBC_g_. Furthermore, to analyze the appropriate application scenario of BC and SIBC, we investigated both changes in the largest connected component (LCC) size and changes in the average travel cost in a network when the central streets (i.e., edges in the Shenzhen street network) or cities (i.e., nodes in China’s intercity network) were attacked.

### Shenzhen street network

Shenzhen is an important economic center of China and has been categorized as an ‘Alpha-’ city (i.e., a first-tier city in the world) by GaWC in 2020 (https://www.lboro.ac.uk/gawc/world2020t.html). The street network here is well-developed and accompanied by heavy traffic. Identifying street segments that play important roles in network structure and functionality can guide road construction and traffic planning in Shenzhen. Moreover, Shenzhen is a strip-shaped polycentric city. Under the influence of urban structure and topography, the structural center of the Shenzhen street network is separated from its functional center. Using this street network as a case study is helpful to highlight the different roles of BC and SIBC so that readers can intuitively understand the different scenarios in which BC and SIBC are appropriate.

The street network used in this case was obtained by digitizing the road layer of Amap. Some simplified processing was carried out on the street network without losing the topological characteristics: (1) multiple lanes were condensed into single lanes, and (2) ramps were incorporated into main roads. The simplified network is treated as an undirected network. Nodes in the network are street intersections, and edges correspond to street segments between intersections. The network includes 1,458 nodes and 2,400 edges, and it is an edge-weighted network where the edge length represents the travel cost of the street segment. To calculate SIBC_o_ and SIBC_g_, the observed number of spatial interactions between each node pair and the node population were obtained by aggregating mobile phone location data into the 500m buffers of nodes. The mobile phone data recorded the populations at different locations and the OD flows between different locations.

**Fig 2:**
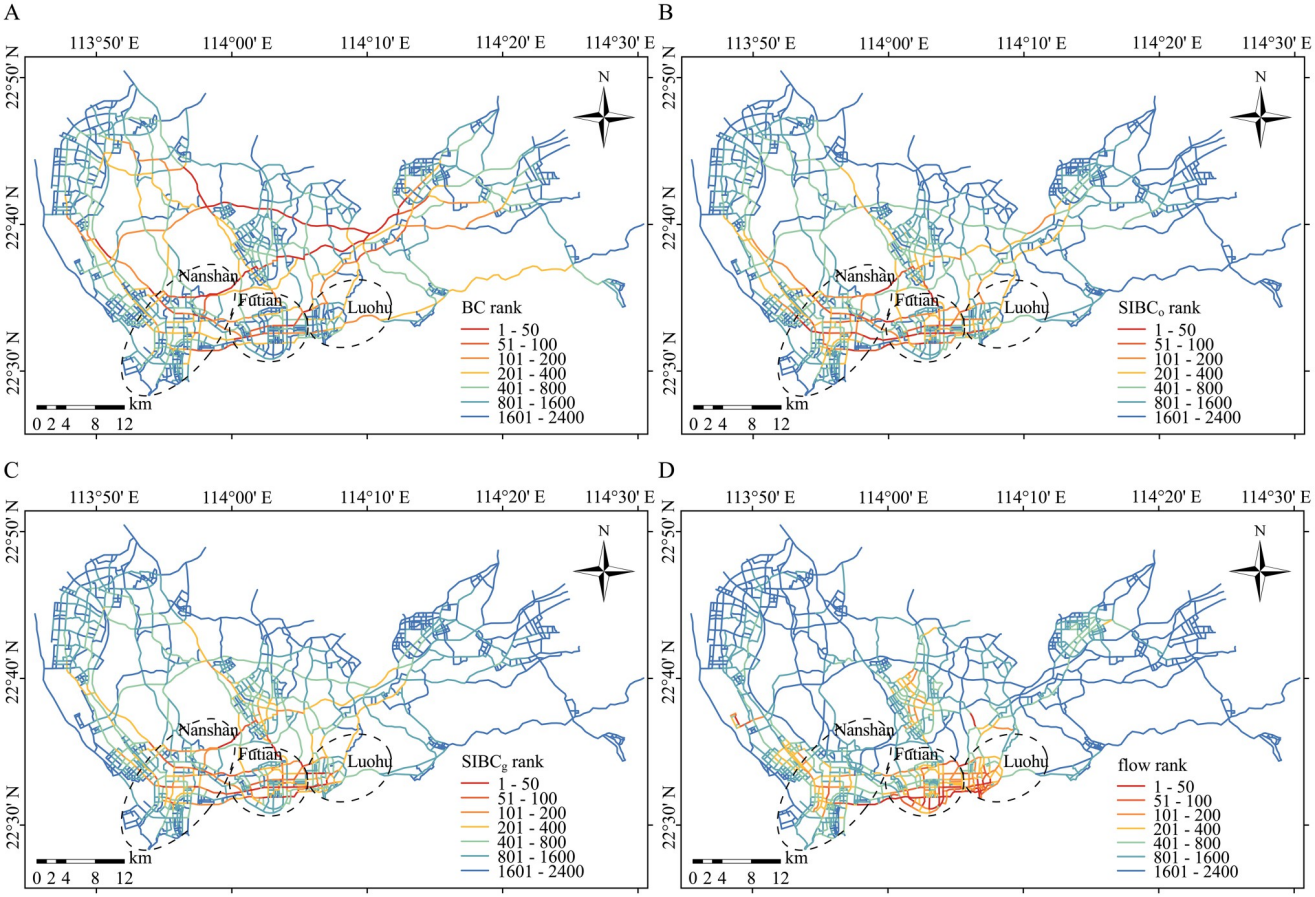
The betweenness centrality of streets in Shenzhen street network (The street network was obtained by manually digitizing, which has been simplified with no copyright disputes). (A) The spatial distribution of the BC rankings. (B) The spatial distribution of the SIBC_o_ rankings. (C) The spatial distribution of the SIBC_g_ rankings. (D) The spatial distribution of the traffic flow rankings.

Fig 2A–2C show the centrality rankings for street segments evaluated using BC, SIBC_o_, and SIBC_g_ (*β* = 1.5 [41]), respectively. The street segments with high BC are mainly located in the city center (Fig 2A) because many of the shortest paths between the northeastern nodes and the western nodes pass through these streets. However, in many application scenarios, such as transport planning and road traffic jam resolutions, it is crucial to know the traffic volume along each street. Since many shortest paths through an edge does not equate to a large traffic volume along it, BC is not applicable to the above scenarios. Compared to the results for BC, the street segments with high SIBC are concentrated in the south of the city—an area that encompasses the three most prosperous districts of Shenzhen: Luohu, Nanshan, and Futian (Fig 2B and 2C). In these districts, high levels of commerce and dense populations lead to active population movements, generating large traffic volumes. Therefore, many people rely on the streets in these areas. If these streets are closed, traffic flows can be greatly disrupted. For application scenarios where traffic volumes along streets are important, central streets identified using SIBC are more applicable than those identified using BC.

**Fig 3:**
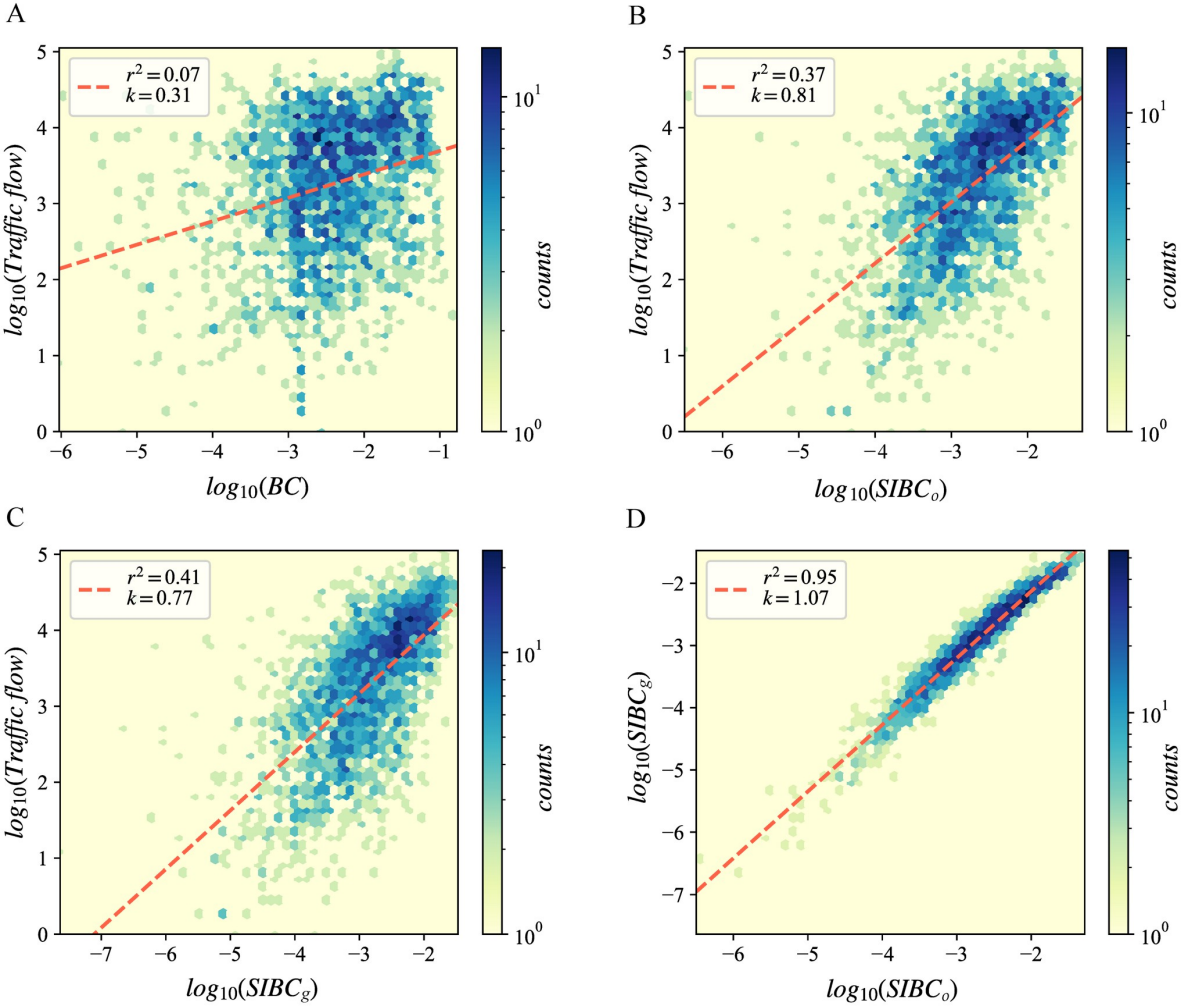
The correlation between traffic flow and the betweenness centrality of streets. (A) The correlation between traffic flow and BC. (B) The correlation between traffic flow and SIBC_o_. (C) The correlation between traffic flow and SIBC_g_. (D) The correlation between SIBC_o_ and SIBC_g_. The colors represent the counts of scatters.

To further quantify the extent to which SIBC is more suitable for identifying critical nodes and edges than BC in spatial networks, we analyze the correlation between the importance of nodes and BC measures. Considering that the traffic volume through a street can reflect the importance of a street in the network functionality, we take it as a reference for evaluating the ability of different betweenness measures to assess the centrality of streets. Specifically, scatter plots and Spearman correlation coefficients for the three BC measures and the street traffic volume were drawn and calculated. The street traffic volume was obtained by matching taxi trajectories to the street network (Fig 2D). The scatter plots in Fig 3A–3C show the correlations between centrality values and traffic volume. The correlation between SIBC_o_ or SIBC_g_ and the traffic volume is strong while that between BC and the traffic volume is weak, demonstrating that SIBC is more applicable to street network analysis than BC in terms of the transportation functionality of a network. In addition, since many applications tend to focus on centrality rankings rather than absolute values, we calculated the Spearman correlation coefficients. The Spearman correlation coefficients between the traffic volume and BC, SIBC_o_, and SIBC_g_ are 0.27, 0.62, and 0.67, respectively, which also shows that SIBC is to a great extent more suitable than the BC for evaluating the centrality of edges in a spatial network. Besides, the scatter plot in Fig 3D shows a strong linear correlation between SIBC_o_ and SIBC_g_, with the slope of the fitted line being close to 1. The Spearman correlation coefficient between SIBC_o_ and SIBC_g_ reaches 0.98. Both results indicate that SIBC_g_ can be a substitute for SIBC_o_ when the actual OD flow matrix is unavailable.

Street networks are key factors determining the form and function of a city and play an essential role in transporting goods, people, and information. It is necessary to consider spatial interactions, rather than simply the network structure when evaluating the centrality of streets for measuring their transportation capacities. The above results indicate that it is sound and valid to introduce variations in spatial interactions into BC for the transportation functionality analysis of street networks. As a by-product, SIBC can estimate the traffic volume on street segments based on the number of spatial interactions between each node pair, which has been verified in previous studies [22–24].

It is worth noting that we have not considered the boundary effect in the above case, so the BC and SIBC actually reflect the centrality of street segments in Shenzhen’s internal street network. That is, the central edges identified by BC and SIBC are specific to the current street network. When the network is expanded to cover the whole Guangdong province or China, the central street segments would change. The similar boundary effect exists for all network indicators. Nevertheless, some researchers have uncovered that the distance decay and the cohesion strength in administrative regions can weaken the boundary effect in spatial network analysis [42]. Therefore, it should be reasonable to select the street network within Shenzhen, which can reduce the influence of the boundary effect to a large extent. In addition, although the results of BC and SIBC would vary depending on the network scope, differences in the principles and roles of BC and SIBC in the centrality assessment should not change. BC still identifies the central edges based on the topology, while SIBC identifies the central edges by combining topology and spatial interaction. In theory, the boundary effect should not affect the conclusion that SIBC is more suitable for network functional analysis than BC. In the future, we can further empirically explore how the boundary effect affects the results of BC and SIBC, but this is not the focus of this study.

### China’s intercity network based on railway

**Fig 4:**
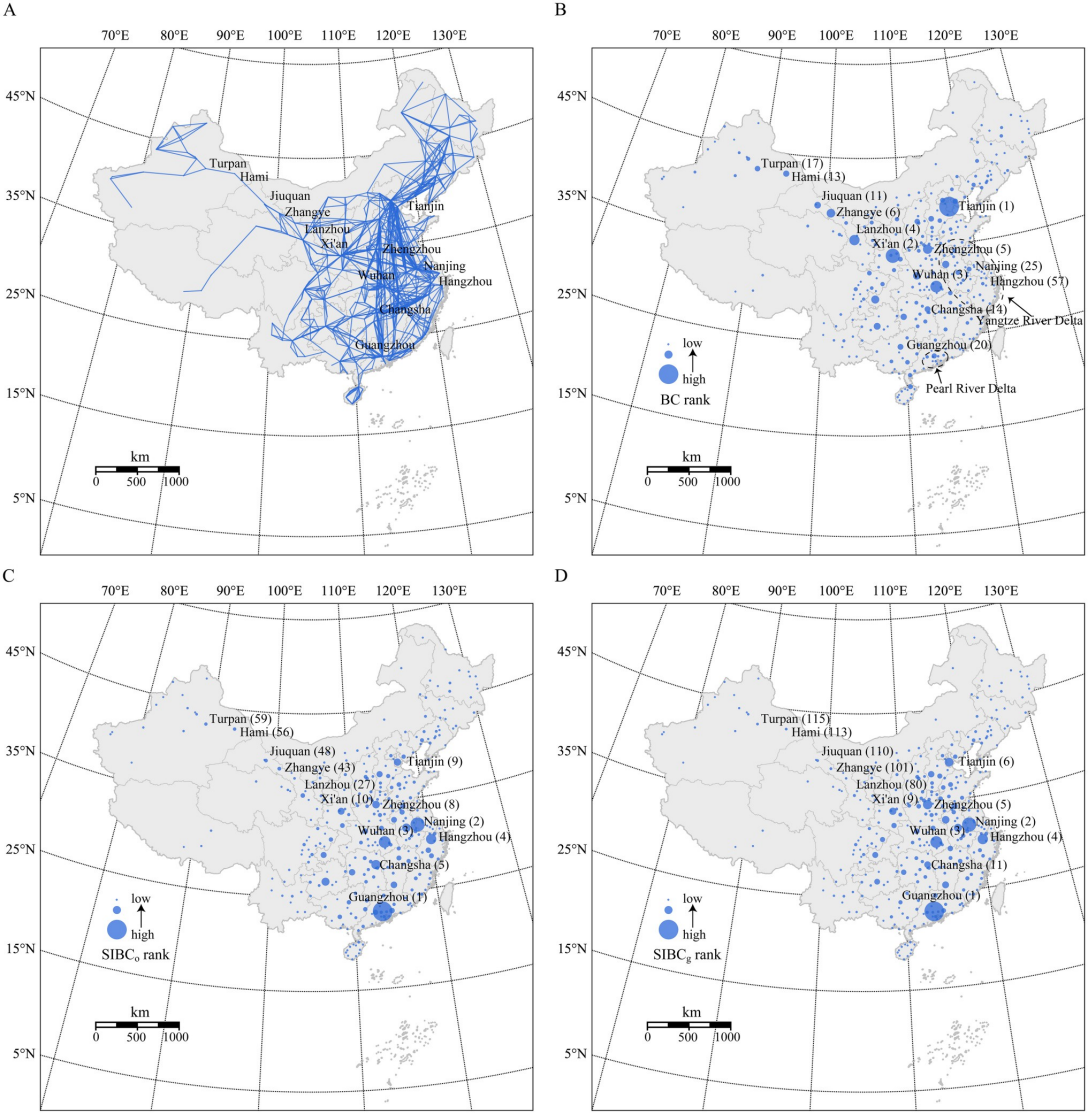
The betweenness centrality of China’s cities (The base map was republished from https://www.resdc.cn/data.aspx?DATAID=200 under a CC BY license, with permission from the Resource and Environment Science and Data Center, Institute of Geographic Sciences and Natural Resources Research, CAS, original copyright 2015. (A) The topological structure of China’s intercity network. (B) The spatial distributions of BC rankings. (C) The spatial distributions of SIBC_o_ rankings. (D) The spatial distributions of SIBC_g_ rankings. The numbers in parentheses correspond to the centrality rankings of the cities.

China’s intercity network was constructed according to 2017 train schedule data. Initially, the train stations in each city were merged into a single node. The *L* space model was then adopted to construct the network, which meant that only two adjacent cities were connected by an edge when a line passes through multiple cities [39]. The resulting network is shown in Fig 4A, where nodes denote cities with at least one train station in their administrative regions and edges connect two adjacent cities on the same rail line. Because data for Hong Kong, Macau, and Taiwan are unavailable, these cities are not included in this network. This network includes 328 nodes and 1,069 edges. The edge cost is determined using the distance between adjacent cities. Because train lines are usually symmetrical, we assume this network is undirected and edge-weighted. The actual number of spatial interactions between cities for SIBC_o_ is based on Baidu migration data. The number of mobile phone users represents the city population in the SIBC_g_.

Fig 4B–4D show the spatial distribution of centrality rankings evaluated using BC, SIBC_o_, and SIBC_g_ (*β* = 1 [43]), respectively. The cities with high BC are mainly distributed in the central and northwestern regions (Fig 4B). In this case, the most central city is Tianjin, followed by Xi’an, Wuhan, Lanzhou, Zhengzhou, and Zhangye. It is not surprising that Wuhan and Zhengzhou, as essential transportation hubs in central China, have high BC rankings, but the importance of Lanzhou and Zhangye seems to defy common sense. Train stations in these two cities are small and are passed through by few trains. Additionally, the population sizes and economic levels of these two cities are limited. The fundamental reason for this result is that BC only considers the network structure. For the network structure, the Lanzhou–Xinjiang railway is a ‘bridge’ between the eastern cities and the cities of Xinjiang Province, so the cities located on that line have high BC rankings. Besides Lanzhou and Zhangye, Jiuquan, Hami, and Turpan all rank in the top 20 for BC. However, for cities located in the Yangtze River Delta and the Pearl River Delta (i.e., two areas with dense railway lines, rapid economic development, and active population movements), the BC rankings are low. This result indicates that central nodes identified using BC mainly act as ‘bridges’ in a network structure.

Fig 4C and 4D show the effect of introducing spatial interactions into BC. After considering the observed OD flow matrix, cities with high centrality are mainly concentrated in the central and eastern regions (Fig 4C). The most central city is Guangzhou, followed by Nanjing, Wuhan, and Hangzhou. Guangzhou and Nanjing are located in economically developed and densely populated areas. The shortest paths passing through them are not the most numerous, but they carry considerable passenger flows, so their SIBC_o_ rankings are high. In contrast to the results for BC, the SIBC_o_ rankings of cities on the Lanzhou–Xinjiang railway line are relatively low because fewer passengers use the Lanzhou–Xinjiang railway. Although the number of paths passing through cities on the Lanzhou–Xinjiang railway is large, the influence of these paths on the centrality of these cities is weakened after weighting these paths with small passenger flows. Fig 4D depicts the results for SIBC_g_ with a similar distribution to Fig 4C. The most central city is still Guangzhou, followed by Nanjing, Wuhan, and Hangzhou. The Spearman correlation coefficient between SIBC_o_ and SIBC_g_ is 0.97, proving that SIBC_g_ can reasonably substitute for SIBC_o_ to introduce spatial interactions into BC.

The comparison between BC and SIBC confirms that the critical nodes identified by SIBC are jointly determined by the number and the weight (the intensity of the spatial interaction) of the shortest paths passing through it. Combining the network structure and spatial interactions can reflect the importance of a node in terms of transportation functionality. Therefore, SIBC can provide a more reasonable assessment of the importance of a city in application scenarios where the distribution of spatial interactions in a network is crucial. For example, when faced with the spread of disease or a Spring Festival travel rush, the control and management of Guangzhou’s railway stations should have a more significant effect on the efficiency of the whole network than that of Lanzhou’s railway stations.

### Robustness analysis for network structure and function

To investigate the application performance of SIBC, we apply it to the robustness analysis of the Shenzhen street network and China’s intercity network. The disruption of different nodes or edges in a network often has different effects on the structure and functionality of the network. Heavy losses occur when critical nodes or edges are disrupted. Network robustness measures the damage to a network after it is disrupted. Most existing robustness studies have focused on the damage to network structures with BC often being used to analyze network structure robustness [44, 45]. The most common method is to delete edges one by one according to the descending order of BC, and then observe the size change of the LCC of the network. In our study, we emphasized the importance of spatial interactions in spatial network analysis. Therefore, in the robustness analysis, we mainly investigated changes in the average travel cost for the LCC of a network with the elimination of critical nodes or edges. The average travel cost was defined as follows:

$$\begin{array}{c} ATC=\frac{\sum_{s,t\in V_{lcs};s\neq t}f\left(s,t\right)\times d_{s,t}}{\sum_{s,t\in V_{lcs};s\neq t}f\left(s,t\right)} \end{array}$$ (9)

where *V*_*lcs*_ represents the node set of the LCC of a network. As stated above, *f*(*s*, *t*) denotes the intensity of spatial interaction between *s* and *t*. For Eq 9, *f*(*s*, *t*) = *O*_*s*,*t*_ when SIBC is calculated in terms of SIBC_o_; *f*(*s*, *t*) = *m*_*s*_ *m*_*t*_/*d*_*st*_ when SIBC_g_ represents SIBC.

**Fig 5:**
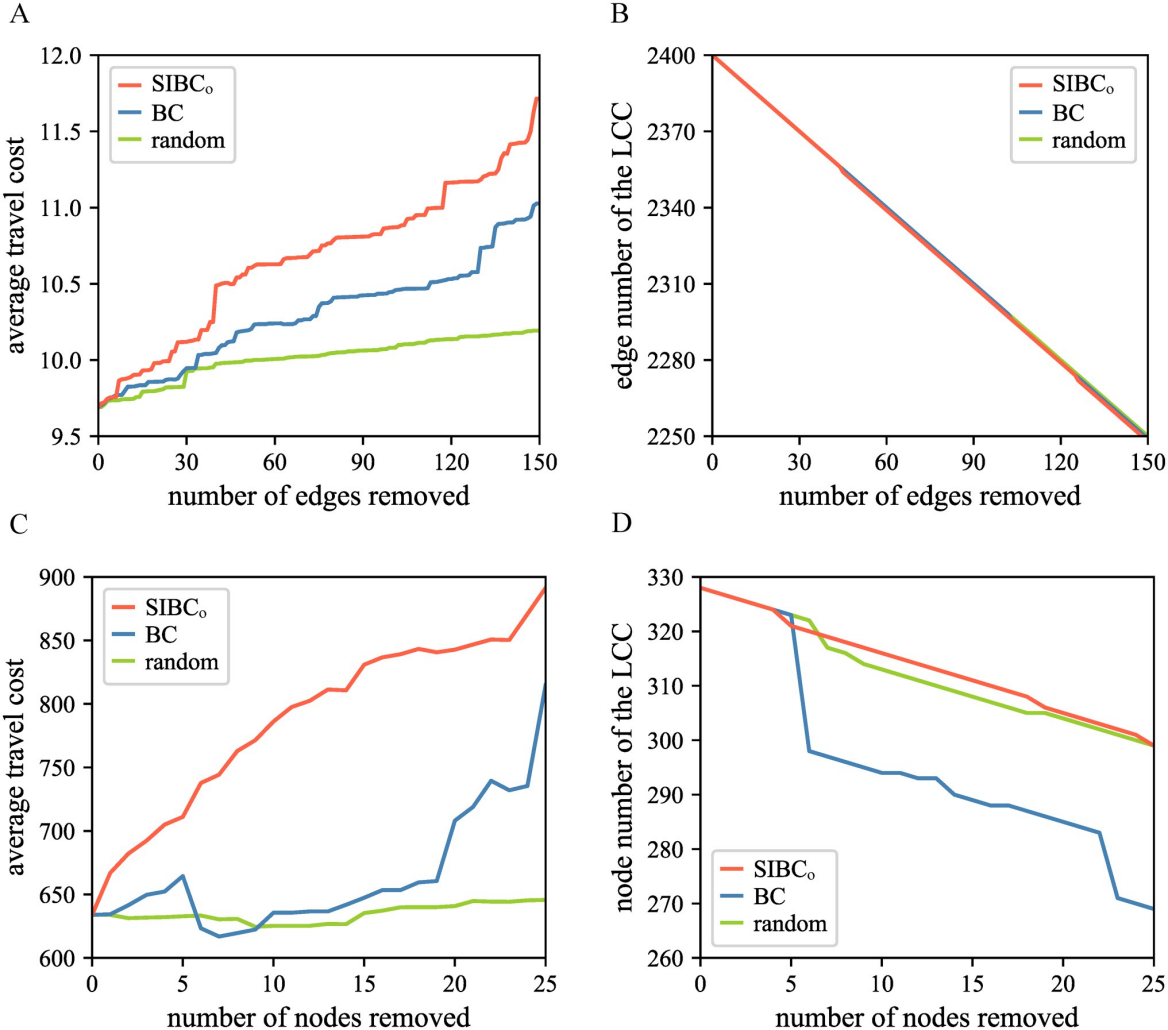
The robustness analysis of two networks. (A) Changes in the average travel cost of the Shenzhen street network as central streets are removed. (B) Changes in the edge number of the LCC as central streets are removed from the Shenzhen street network. (C) Changes in the average travel cost of China’s intercity network as central cities are removed. (D) Changes in the node number of the LCC as central cities are removed in China’s intercity network.

Note that, in the robustness analysis shown in Fig 5, the intensity of spatial interaction is represented by the observed OD flow. The robustness analysis using SIBC_g_ is shown in S1 Fig. Since the results in S1 Fig are similar to that in Fig 5, we use the general expression SIBC in the analysis below.

In street networks, traffic congestion, road maintenance, urban marathons, and other factors often put some street segments out of action, leading to some people have to choose alternative travel routes. Such changes in routes tend to increase travel costs. The more important the damaged street segment, the more the costs increase. To compare central streets identified by which measure is more crucial for the network functionality, we explored the effect of removing streets with high BC or high SIBC on the average travel cost of the network. Streets were sorted in descending order according to BC or SIBC, and then the top 150 streets were removed one by one. Each time a street was removed, we redistributed the OD interaction flow in the new network and calculated the corresponding average travel cost. Fig 5A shows how the average travel cost changes as central streets are removed, providing a randomized trial for the comparison. The result demonstrates that when central streets evaluated using the SIBC are eliminated, the average travel cost of the network increases the most. It means that the centrality calculated using the SIBC better reflects the influence of an edge on the network functionality than that calculated using BC. For practical application, keeping streets with high SIBC unblocked can prevent an increase in the average travel costs and a decrease in travel efficiency. Fig 5B shows that the deletion of the top 150 edges for SIBC or BC does not significantly affect the edge number for the LCC, indicating that the network structure is stable. It can be concluded that, after excluding the effect of the network size on the travel cost, the influence of central streets identified using SIBC on the travel cost is greater than that identified using BC.

For China’s intercity network based on the railway, we analyzed the impact of deleting critical cities on the average travel cost and the size of the LCC. After sorting cities according to SIBC or the BC from high to low, the top 25 nodes were removed one by one. Fig 5C shows changes in the average travel cost when central cities are removed. The average travel cost increases the most when cities with high SIBC are removed, indicating that central cities identified using the SIBC have a greater impact on the network’s transportation functionality than those identified using BC. Fig 5D shows that removing central cities identified using BC has a greater effect on the node number of the LCC than removing those identified using SIBC. This result shows that BC is more focused on the influence of a node on the network structure than SIBC. Therefore, evaluating the centrality of a node using SIBC can yield more valuable results than using BC for application scenarios where the network functionality is concerned. For example, the improved inspection and maintenance of railway tracks in cities with high SIBC could reduce the risk of accidents. For the prevention and control of an epidemic, railway stations in cities with high SIBC should be monitored and checked more intensively.

In the robustness analysis of two networks, removing critical nodes or edges identified by SIBC has a more significant impact on average travel costs than removing those identified by BC. The different performance of BC and SIBC in robustness analysis indicates that SIBC can help improve network travel efficiency, while BC focuses on measuring the vulnerability of network topology. Therefore, besides traffic flow estimate and hub node identification, SIBC can be applied effectively in robustness analysis for network functionality.

## Discussion

In the previous sections, the idea of introducing spatial interaction into BC for spatial networks has been illustrated in theory and applications. Is the philosophy behind the modified measures just for spatial networks? It would be instructive to discuss the rationality and feasibility of applying SIBC to social networks.

In social networks, the amount of information transmitted between two nodes are often difficult to characterize. Nevertheless, node attributes and the network distance between two nodes are easy to define, which makes it practical to generalize the gravity model incorporated betweenness centrality to social networks. As mentioned in 2.3, the gravity model incorporated betweenness centrality can make the concept of SIBC more fundamental. Node attributes and the network distance can influence the centrality of nodes or edges separately. This idea also applies to common social networks. In social networks, node attributes can reflect the communication ability or status of corresponding persons, which have a positive influence on the centrality of nodes or edges. The distance between two nodes can affect the time cost or the amount of information loss in the communication, which has a negative effect on the centrality of nodes or edges. It is common sense that if two “big shots” are connected via a third person, then the person is more important than the person who connects two average individuals. Therefore, both the node attribute and the communication distance should be helpful to understand the importance of nodes or edges in a social network.

**Fig 6:**
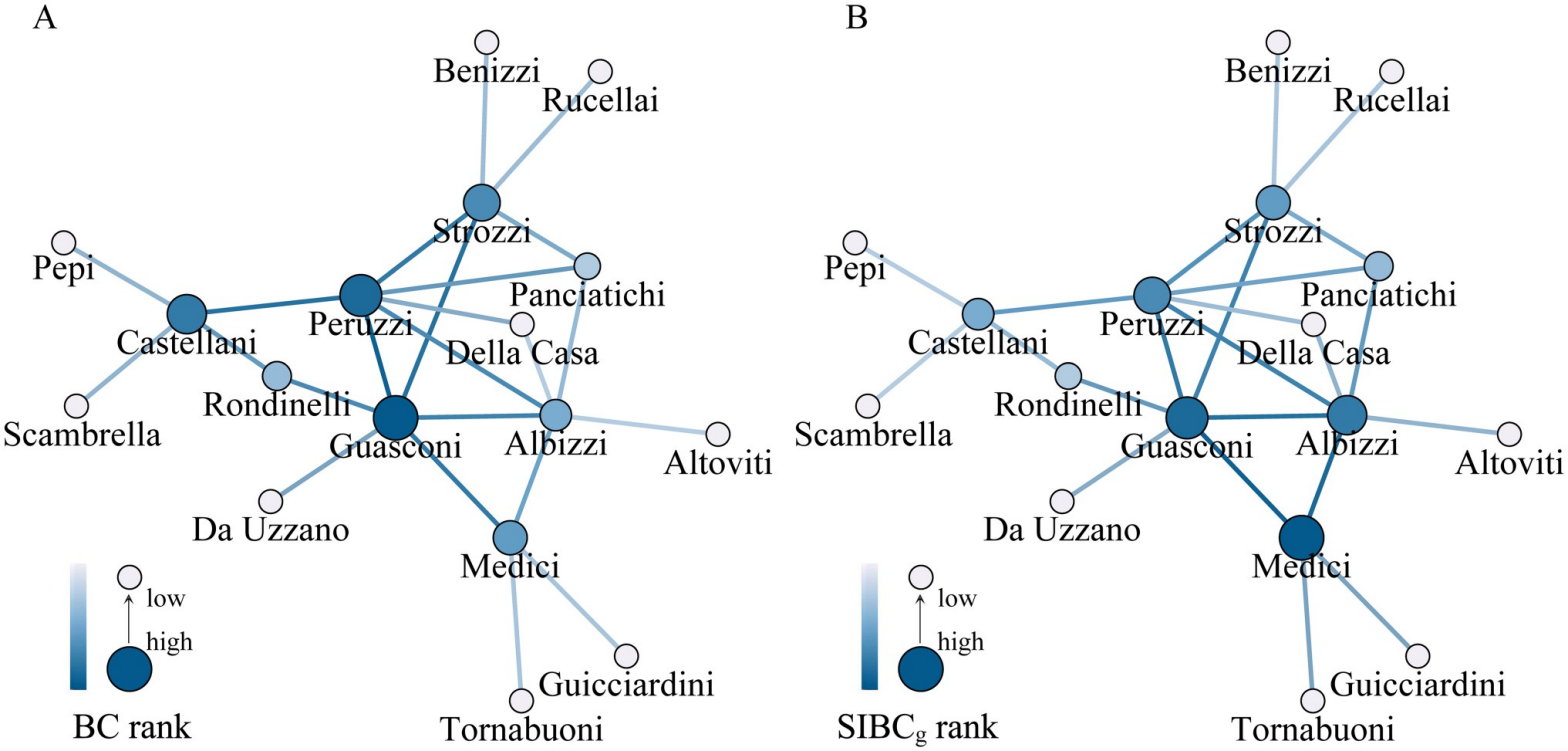
The betweenness centrality of Florence families. (A) The BC rankings for Florence family’s network. (B) The SIBC_g_ rankings for Florence family’s network. Each node denotes a family, whose size and color represent the centrality ranking of the corresponding node. The colored lines denote the centrality rankings of edges. The gross wealth of families are listed as follow: Albizzi: 249,940; Altoviti: 77,421; Benizzi: 26,093; Castellani: 111,355; Da Uzzano: 96,131; Della Casa: 140,624; Guasconi: 341,198; Guicciardini: 203,087; Medici: 248,105; Panciatichi: 193,878; Pepi: 43,100; Peruzzi: 150,375; Rondinelli: 43,588; Rucellai: 93,891; Scambrilla: 148; Strozzi: 407,296; Tornabuoni: 299,878.

To verify the effectiveness of generalizing the gravity model incorporated betweenness centrality into spatial networks, we apply it to the Florence family’s network. The Florence family’s network has been used as an empirical case study in many studies about centrality [20, 46, 47]. It is an unweighted network containing 17 Renaissance Florentine families as nodes and 23 marriages as edges. In the computation of the gravity model incorporated betweenness centrality, path lengths are measured by the topological distance. Node attributes are represented by the gross wealth quantified in florins [48], which are listed in the caption of Fig 6.

Fig 6A shows that the most central family is the Guasconi family and the most influential edge corresponds to the marriage between the Guasconi family and the Peruzzi family. However, this result is inconsistent with the record in all history books that the Medici family was the most important family in the early fifteenth century in Florence [20]. Therefore, it can be concluded that the conventional BC is not enough to reveal the statuses of different families in the Florence family’s network. Considering the gross wealth and the topological distance, Fig 6B shows that the Medici family becomes the most central, and the marriage between the Medici family and the Guasconi family is the most important. This result demonstrates that the Medici family has the greatest influence on the socioeconomic structure of the Florence families, which is coherent with the historical record. Although the Medici family is not the richest (ranking 5th), it connects four of the five richest families (excluding the Medici). Moreover, the SIBC_g_ ranking of the Albizzi family is also higher than its BC ranking, which is because many families rely on it to connect with four of the five richest families (excluding the Albizzi). Therefore, it is meaningful to combine the topology structure with the node attribute and the communication distance when identifying critical nodes and edges in such a social network.

The effective application of the gravity model incorporated betweenness centrality in the Florence family’s network can provide a good inspiration: the centrality of nodes or edges in a social network can be reasonably evaluated if a suitable definition of node attributes and path lengths is available. Furthermore, this case illustrates that the role of SIBC_g_ in measuring the importance of nodes and edges can be independent of the estimation of interaction intensities. Therefore, SIBC_g_ is not only a representation for SIBC but also makes the concept of SIBC more general.

## Conclusion

BC has attracted much attention in network sciences. To make BC more suitable for spatial networks, we propose SIBC that combines the network structure with variations in spatial interactions. In SIBC, the shortest path between each node pair is weighted by the intensity of spatial interactions between them, leading to different paths contributing differently to the centrality of nodes or edges. To comprehensively understand the impact of spatial interaction on the ability of BC to identify critical nodes and edges, we explored two specific forms of SIBC: SIBC_o_ and SIBC_g_.

To investigate the validity of SIBC_o_ and SIBC_g_, we applied them to the Shenzhen street network and China’s intercity network. In both networks, the spatial distribution of centrality rankings showed the different focuses of BC and SIBC: The central nodes or edges identified by BC often act as ‘bridges’ in the network structure; the central nodes or edges identified by SIBC tend to be located in densely populated and economically developed areas. For the street network, the centrality of a street was proportional to the traffic volume along it, and the correlation between the SIBC and the traffic volume was stronger than that between the BC and the traffic flow. It can be concluded that SIBC can provide more practical and significant results than BC when both the network structure and the network functionality are considered, while BC identifies central nodes or edges only focusing on the network structure. Moreover, the correlation between SIBC_o_ and SIBC_g_ was close to 1 in both networks, proving that the SIBC_g_ can be a valid alternative to the SIBC_o_. The different calculation and data requirements of SIBC_o_ and SIBC_g_ ensure that the idea of incorporating spatial interactions into BC can be applied to many application scenarios. In the robustness analysis, critical nodes and edges identified using SIBC mainly affect the average travel cost in a spatial network, proving the application value of SIBC. Moreover, we discussed the extension of SIBC into social networks. The most critical family identified by SIBC_g_ in the Florence family’s network is consistent with the historical record, implying that it is meaningful to generalize SIBC to social networks.

The major contributions of this study are summarized as follows:

1. We provide a general SIBC measure to identify critical nodes and edges in spatial networks. Compared with BC, SIBC is more suitable for evaluating the centrality of nodes or edges in a spatial network when the network functionality is considerable.
2. We discover that central nodes or edges identified using SIBC have a significant impact on the average travel cost in a spatial network. This demonstrates that the application of SIBC in robustness analysis can provide useful guidelines for improving network efficiency and controlling travel costs.
3. We propose SIBC_g_ as a representation of SIBC, which has a more accessible data demand than SIBC_o_ and makes the concept of SIBC more fundamental. SIBC_g_ can extend the idea of introducing the spatial interactions into BC to wider application scenarios. Its effective application in the classic social network can provide an unusual inspiration for future research on network centrality.

This study suggests the significance of providing a definitive explanation of the spatial interaction in the empirical models of complex networks. We expect the proposed measures to be applied to various spatial problems, such as transportation planning, disease transmission, and resource allocation. Further research is required to introduce more spatial elements and laws, such as spatial dependence and spatial heterogeneity, into various network analysis methods. It can be foreseen that a series of methods and indicators considering spatial characteristics will provide more meaningful and valuable findings for spatial networks.

In the future, our measure can be improved from the following three aspects: First, our measure still assumes that spatial interactions follow the shortest path, but this assumption is not applicable to all networks. To make the measure more applicable, it is workable to combine our idea of weighting paths by the number of spatial interactions with some existing BC variants that consider non-shortest paths. For example, paths could be chosen by random walking and then weighted by the number of spatial interactions. Second, we consider only two forms to represent the spatial interaction. In fact, other spatial interaction models at the population level, such as the intervening opportunity model [49] and the radiation model [50], can also be applied to estimate the interaction intensity. In various application scenarios, it is logical to choose a suitable model to simulate the spatial interaction. Third, our measure cannot adjust the degree of effect of the network structure or spatial interactions on BC values. The measure only considers that spatial interactions play a role in evaluating centrality along with the topology but cannot quantify the effect of these two factors separately.

## Supporting information

S1 Fig The robustness analysis using the gravity model incorporated betweenness centrality. (A) Changes in the average travel cost of the Shenzhen street network as central streets are removed. (B) Changes in the edge number of the LCC as central streets are removed from the Shenzhen street network. (C) Changes in the average travel cost of China’s intercity network as central cities are removed. (D) Changes in the node number of the LCC as central cities are removed in China’s intercity network.
(PDF)
