# Supplementary material for: A spatial interaction incorporated betweenness centrality measure
Source: PLoS One. 2022 May 20;17(5):e0268203. doi: 10.1371/journal.pone.0268203 (PMC9122268; doi:10.1371/journal.pone.0268203)
Supplement: S1 Fig — (A) Changes in the average travel cost of the Shenzhen street network as central streets are removed. (B) Changes in the edge number of the LCC as central streets are removed from the Shenzhen street network. (C) Changes in the average travel cost of China’s intercity network as central cities are removed. (D) Changes in the node number of the LCC as central cities are removed in China’s intercity network. (PDF) [file pone.0268203.s001.pdf]

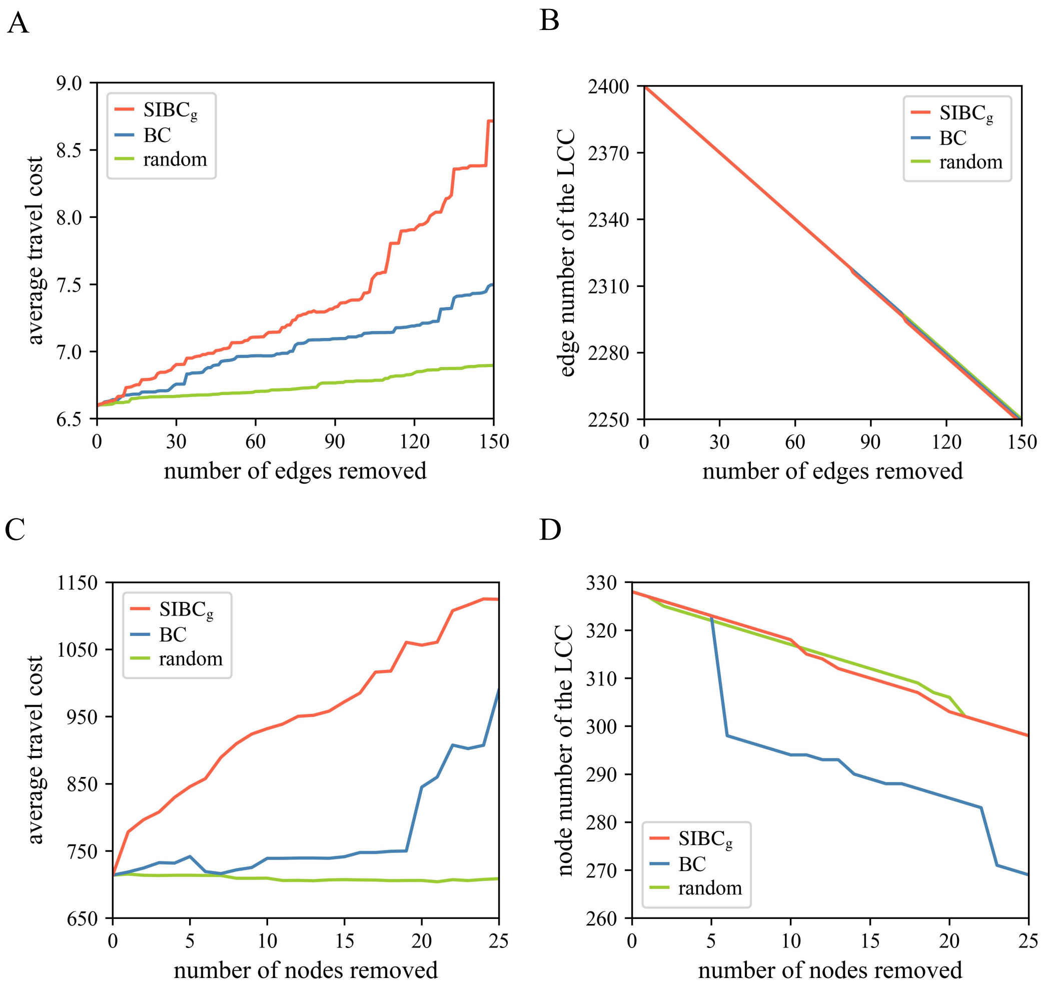

S1 Fig. The robustness analysis using the gravity model incorporated betweenness centrality. (A) Changes in the average travel cost of the Shenzhen street network as central streets are removed. (B) Changes in the edge number of the LCC as central streets are removed from the Shenzhen street network. (C) Changes in the average travel cost of China's intercity network as central cities are removed. (D) Changes in the node number of the LCC as central cities are removed in China's intercity network.
